# Effect of replacing commonly consumed fruit in the United States with berries in the USDA healthy Dietary Patterns: a modeling analysis

**DOI:** 10.1080/07853890.2025.2517817

**Published:** 2025-06-12

**Authors:** Kim S. Stote, Kristi Crowe-White, Leslie Wada

**Affiliations:** a State University of New York, Empire State University, Albany, NY, USA; b The University of Alabama, Tuscaloosa, AL, USA; c North American Blueberry Council, Folsom, CA, USA

**Keywords:** Berries, US department of agriculture Dietary Patterns, food pattern modeling, anthocyanins

## Abstract

**Introduction:**

A recent review of the evidence suggests that berry consumption may have an effect in reducing cardiovascular disease risk.

**Objective:**

To assess the nutritional impact of replacing a serving of commonly consumed fruit with a serving of berries in the US Department of Agriculture (USDA) Dietary Patterns: Healthy US-Style (HUS), Healthy Mediterranean-Style (HMS), and Healthy Vegetarian (HV).

**Methods:**

Using the USDA’s food pattern modeling approach, three 7-day replacement menus were developed for the HUS, HMS, and HV patterns at the 2000-calorie level. The menus included one serving of mixed berries replacing one serving of commonly consumed fruits (apples, bananas, or grapes) while meeting recommendations for other food groups. A total of 2 servings of fruit per day were used in the menus. Nutrient and anthocyanin analyses were conducted on the 7-day menus using ESHA Food Processor: 11.14.x software and the USDA Database for Flavonoid Content of Selected Foods, Release 3.3.

**Results:**

Replacing a serving of commonly consumed fruit with a serving of berries per day in the 2000 calorie USDA Dietary Patterns resulted in an increase of anthocyanins by 93.8%, with *a* ≤ 2.0% increase or decrease in calories, macronutrients, fatty acids, and cholesterol. Dietary fiber increased by 4.7% in the HUS and 5.1% in the HMS USDA Dietary Patterns with minimal increases (< 1.5%) in the HV USDA Dietary Patterns. Soluble fiber increased by 10.1%, 5.3%, and 10.2% in the HUS, HMS, and HV USDA Dietary Patterns, respectively. Vitamin C increased by 16% in the HUS, 13.9% in the HMS, and 11.9% in the HV USDA Dietary Patterns.

**Conclusion:**

The addition of berries to the USDA Dietary Patterns increased beneficial dietary components and nutrients with minimal change in calories, macronutrients, fatty acids, and cholesterol.

## Introduction

As part of the 2025–2030 Dietary Guidelines for Americans (DGA) process, the United States (US) Department of Health and Human Services (HHS) and US Department of Agriculture (USDA) released a list of proposed scientific questions for public comment that will inform the work of the Dietary Guidelines Advisory Committee (DGAC). A key question of focus is: should changes be made to the USDA Dietary Patterns, Healthy US-Style (HUS), Healthy Mediterranean-Style (HMS), and/or Healthy Vegetarian (HV), and should additional USDA Dietary Patterns be developed/proposed based on findings from systematic reviews, data analysis, and/or food pattern modeling analyses, noting that changes to USDA Dietary Patterns may include increases or decreases in amounts of food groups/subgroups and/or recategorization of food groups/subgroups [1]?

Currently, the vegetable group in the DGA is divided into subgroups with specific intake recommendations by color designations (e.g. dark green, orange, red, and yellow) and type (e.g. starchy vegetables, beans, peas, and lentils) to promote nutrient adequacy [2]; however, the fruit group is not segmented. Like vegetables, fruit also provides a diverse range of nutrients and polyphenols based on their color and type, suggesting that a more nuanced classification for the fruit group may be warranted [2]. For example, berries (including blackberries, blueberries, raspberries, and strawberries) are uniquely high in anthocyanins that are responsible for their vibrant blue and red color. In addition, berries contain vitamin C and fiber and are low in calories, which may benefit cardiometabolic health [3]. A recent review of the evidence suggests that anthocyanin-rich foods, such as berries, may be readily incorporated into the diet and is a simple dietary change that could have a significant impact in reducing cardiovascular disease risk [4].

Creating fruit subgroups may encourage greater diversity of fruit intake, nutrients, and dietary components. The USDA Dietary Patterns include fruit, varying by energy level [2]. What We Eat in America, National Health and Nutrition Examination Survey (NHANES) shows that less than half of adults reported fruit intake on any given day and the majority consumed only one fruit, with bananas most consumed, followed by apples and grapes [5]. In addition, the USDA, Economic Research Service, reports that the most common total fruit available for consumption in the US include apples, bananas, and grapes [6]. While these fruits are nutrient dense and offer health benefits, their dominance in the American diet may limit the breadth of nutrients that a more diverse intake could provide. For example, in weekly food patterns, consumption of the recommended amount of fruit intake consisting of solely bananas would not provide as many varied nutrients as a weekly food pattern consisting of berries, citrus fruits (e.g. grapefruit and oranges), and stone fruits (e.g. cherries and peaches). Research shows that consuming a variety of fruit may be associated with greater overall nutrient intake and may promote better adherence to dietary recommendations [7].

The objective of the current study is to assess the nutritional impact of replacing one serving of commonly consumed fruit in the US, such as apples, bananas, and grapes, with a serving of berries per day in the HUS, HMS, and HV USDA Dietary Patterns.

## Methods

The current study is based upon the USDA’s food pattern modeling approach that develops food patterns that meet nutritional goals for people in the US ages 2 years and older [2]. The USDA Dietary Patterns represent the types and amounts of food groups that provide adequate nutrients or dietary components, such as fiber, to meet Dietary Reference Intakes (DRIs) and DGA recommendations at various energy levels by age-sex groups ages 2 years and older. The USDA Dietary Patterns are updated every 5 years during discussions of the DGA Committee and are presented to the DGA Committee for its assessment of how well the USDA Dietary Patterns support current evidence on diet, health, and nutrient adequacy. In addition, the USDA Dietary Patterns provide amounts of foods from the 5 major food groups and subgroups that include fruit; vegetables (dark green, orange, red and yellow, starchy vegetables, beans, peas, and lentils); dairy; grains; and protein foods. The food pattern modeling approach used in the current study identified the appropriate energy level for the foundational menus; determined nutritional goals and established food groupings for the USDA Dietary Patterns; determined the amounts of nutrients and dietary components that would be obtained by consuming various foods within each group; and evaluated nutrient and dietary component levels compared to nutritional goals [8]. Thus, three 7-day replacement menus were developed to reflect where berries are consumed in place of commonly consumed fruit in the HUS, HMS, and the HV USDA Dietary Pattern models for the 2000-calorie level. Since the DGA establishes dietary patterns at 200 calorie intervals (i.e. 1800, 2000, 2200, 2400), menus were kept within ± 100 kcal of 2000 calories; therefore, the calorie levels were rounded to the 2000 calorie pattern (not 1800 or 2200) (Appendix 1, supplementary material).

The replacement menus included 2 servings of fruit per day, per the DGA recommendation for a 2000-calorie level diet and detailed the impact of consuming 7 servings of berries per week and 7 servings of commonly consumed fruits versus 14 servings of commonly consumed fruit. This is similar to how the DGA vegetable groups are currently subdivided into servings per week. One serving of fruit is defined as 1 cup of fruit. The replacement menus incorporated one serving of mixed berries replacing one serving of 1 of the 3 commonly consumed fruit (apples, bananas, and grapes) while adhering to other DGA recommendations for vegetables, dairy, grains, and protein foods. For 3 of the 7 days, 1 cup of blueberries (fresh or frozen) and 4 of the 7 days 1 cup of mixed berries (fresh or frozen, 1/3 cup blackberries, 1/3 cup raspberries, 1/3 cup strawberries) were used. Blueberries were selected because they contain higher levels of anthocyanins compared to the other berries [9]. Nutrient and dietary component profiles for the commonly consumed fruit (apples, bananas, and grapes) and berries were computed for 1 cup equivalent serving (Table 1). Nutrient analyses were conducted on the 7-day menus for the HUS, HMS, and HV USDA Dietary Patterns for the 2000-calorie level using ESHA’s Food Processor: 11.14.x (released October 2023) Nutrition Analysis software. ESHA’s Food Processor Nutrition Analysis software master database includes a comprehensive collection of ingredients, recipes, and other food items. It provides detailed information for each item covering up to 172 data fields, such as proximates (ash, moisture, protein, fat, carbs), vitamins, minerals, and other nutrient components. The data for the master database is gathered from more than 1900 reputable sources, which include the Food and Nutrient Database for Dietary Studies (FNDDS), the most recent USDA Standard Reference database, manufacturer’s data, USDA FoodData Central Brands, and others [10]. Anthocyanin analysis of the menus was determined using the USDA Database for Flavonoid Content of Selected Foods, Release 3.3 [9]. Food costs were obtained in September 2024 from online market prices at Walmart in Grand Junction, Colorado. Walmart’s widespread national presence and standardized pricing make it a practical and accessible source for estimating mean food costs. Grand Junction offers a representative sample of mid-cost US regions, avoiding the extremes of high-cost urban centers and low-cost rural areas [11].

**Table 1.** Nutrient and dietary component profiles of commonly consumed fruit and berries.^a^

	Fresh blueberries (per 1 cup/150 g)	Frozen blueberries (per 1 cup/150 g)	Fresh mixed berries (per 1 cup/150 g)^b^	Frozen mixed berries (per 1 cup/150 g)^b^	Fresh, apple, small (per 1 cup/150 g)	1 large banana	Fresh grapes, green (per 1 cup/150 g)
Macronutrients							
Calories (kcal)	84.36	79.05	56.75	73.12	77.48	121.04	104.19
Protein (g)	1.10	0.65	1.47	1.35	0.39	1.48	1.09
Carbohydrates (g)	21.45	18.86	13.07	17.82	20.58	31.06	27.33
Fiber, total (g)	3.55	4.18	5.97	6.52	3.58	3.54	1.36
Soluble Fiber, total (g)	0.40	1.24	0.71	0.89	0.36	0.97	0.69
Fat (g)	0.49	0.99	0.63	0.56	0.25	0.45	0.24
Fatty acids, total saturated (g)	0.04	0.08	0.03	0.01	0.04	0.15	0.08
Fatty acids, total monounsaturated (g)	0.07	0.14	0.07	0.03	0.01	0.04	0.01
Fatty acids, total polyunsaturated (g)	0.22	0.43	0.35	0.15	0.08	0.10	0.07
Trans fatty acids (g)	0.00	0.00	0.00	0.00	0.00	0.00	0.00
Cholesterol (mg)	0.00	0.00	0.00	0.00	0.00	0.00	0.00
Vitamins							
Vitamin A – RAE (mcg)	4.00	3.56	6.04	4.71	4.02	4.35	4.98
Vitamin B1 – Thiamin (mg)	0.05	0.05	0.03	0.03	0.03	0.04	0.10
Vitamin B2 – Riboflavin (mg)	0.06	0.06	0.04	0.06	0.04	0.10	0.11
Vitamin B3 – Niacin (mg)	0.62	0.81	0.73	1.11	0.14	0.90	0.28
Vitamin B6 (mg)	0.08	0.09	0.05	0.07	0.06	0.50	0.13
Vitamin B12 (mcg)	0.00	0.00	0.00	0.00	0.00	0.00	0.00
Vitamin C (mg)	14.36	3.87	48.55	33.90	6.85	11.83	4.83
Vitamin D – IU (IU)	0.00	0.00	0.00	0.00	0.00	0.00	0.00
Vitamin E (alpha-tocopherol) (mg)	0.84	0.74	1.05	1.12	0.27	0.14	0.29
Folate, total (mcg)	8.88	10.85	31.80	35.00	4.47	27.20	3.02
Vitamin K (mcg)	28.56	25.42	13.63	14.55	3.28	0.68	22.05
Minerals							
Calcium (mg)	8.88	12.40	31.53	33.87	8.94	6.80	15.10
Copper (mg)	0.08	0.05	0.14	0.12	0.04	0.11	0.19
Iodine (mcg)	0.44	0.44	0.19	4.43	0.15	0.41	0.60
Iron (mg)	0.41	0.28	0.76	1.09	0.18	0.35	0.54
Magnesium (mg)	8.88	7.75	24.61	26.53	7.45	36.72	10.57
Phosphorus (mg)	17.76	17.05	33.62	34.74	16.39	29.92	30.20
Potassium (mg)	113.96	83.70	210.98	212.29	159.43	486.88	288.41
Selenium (mcg)	0.15	0.15	0.46	0.63	0.00	1.36	0.15
Sodium (mg)	1.48	1.55	1.37	1.94	1.49	1.36	3.02
Zinc (mg)	0.24	0.11	0.49	0.37	0.06	0.20	0.11
Anthocyanins (mg)	241.68	253.12	81.18	87.24	2.37	0.00	0.00

^a^Values for macronutrients, vitamins and minerals are from ESHA Food Processor: 11.14.x (released October 2023) software; values for anthocyanins are from the USDA Database for Flavonoid Content of Selected Foods, release 3.3.

^b^50 g blackberries, 50 g raspberries and 50 g strawberries.

## Results

Replacing a serving of commonly consumed fruit, such as apples, bananas, and grapes, with a serving of berries per day in the 2000 calorie HUS USDA Dietary Patterns resulted in anthocyanins increasing by 93.8%. There was less than a 2.0% decrease in calories, protein, fatty acids, and cholesterol with a 3.1% decrease in carbohydrates. There was a 5.1% increase in total dietary fiber and a 10.1% increase in soluble fiber. Total sugar decreased by 8.1%. For vitamins there was a 16.0% increase in vitamin C, a 6.0% increase in vitamin E, a 5.4% increase in vitamin K, and less than a 2.0% decrease or increase in other vitamins. For minerals there was a 3.3% decrease in potassium and less than a 2.0% decrease or increase in other minerals. Cost differential was an increase of $7.28 per week, which is a 10.4% increase (Table 2).

**Table 2.** Effect of replacing a serving of commonly consumed fruit with a serving of berries per day on calories and nutrients in the 2000 calorie USDA dietary pattern healthy US-Style.^a^

	2000 kcal Healthy US-Style	2000 kcal Healthy US-Style with berry replacement
Macronutrients		
Calories (kcal)	2017.77	1991.98
Protein (g)	114.39	114.67
Carbohydrates (g)	257.77	249.75
Fiber, total (g)	34.18	35.93
Soluble Fiber, total (g)	2.83	3.12*
Fat (g)	64.51	64.83
Fatty acids, total saturated (g)	14.91	14.86
Fatty acids, total monounsaturated (g)	25.64	25.70
Fatty acids, total polyunsaturated (g)	18.52	18.75
Trans fatty acids (g)	0.16	0.16
Cholesterol (mg)	274.06	274.06
Vitamins		
Vitamin A – RAE (mcg)	1022.91	1023.33
Vitamin B1 – Thiamin (mg)	1.63	1.63
Vitamin B2 – Riboflavin (mg)	2.87	2.84
Vitamin B3 – Niacin (mg)	24.66	24.96
Vitamin B6 (mg)	2.79	2.66
Vitamin B12 (mcg)	6.29	6.29
Vitamin C (mg)	124.13	143.95*
Vitamin D – IU (IU)	424.39	424.39
Vitamin E (alpha-tocopherol) (mg)	11.32	12.00
Folate, total (mcg)	515.55	523.99
Vitamin K (mcg)	256.42	270.23
Minerals		
Calcium (mg)	1283.35	1292.35
Copper (mg)	1.94	1.94
Iodine (mcg)	259.37	259.29
Iron (mg)	18.46	18.68
Magnesium (mg)	497.83	496.59
Phosphorus (mg)	2181.81	2182.07
Potassium (mg)	4182.45	4043.83
Selenium (mcg)	135.95	135.80
Sodium (mg)	2027.47	2027.02
Zinc (mg)	13.75	13.97
Anthocyanins (mg)	11.55	185.85*
Food cost ($)	70.16	77.44

^a^The 7 day berry replacement menu included 2 servings of fruit per day for a 2,000-calorie level with 7 servings of berries per week and 7 servings of commonly consumed fruit (apples, bananas, grapes) versus 14 servings of commonly consumed fruit in the USDA Dietary Pattern Healthy US-Style. * > 10% increase or decrease from the USDA Dietary Pattern Healthy US-Style.

Replacing a serving of commonly consumed fruit, such as apples, bananas, and grapes, with a serving of berries per day in the 2000 calorie HMS USDA Dietary Patterns resulted in anthocyanins increasing by 93.8%. There was a 3.0% or less decrease or increase in calories, macronutrients, fatty acids, and cholesterol. There was a 4.7% increase in total dietary fiber and a 5.3% increase in soluble fiber. Total sugar decreased by 8.1%. For vitamins there was a 13.9% increase in vitamin C, a 5.3% increase in vitamin E, a 4.7% increase in vitamin K, and less than a 3% increase or decrease in other vitamins. For minerals there was less than or equal to 2.0% increase or decrease in other minerals. Cost differential was an increase of $6.72 per week, which is a 9.2% increase (Table 3).

**Table 3.** Effect of replacing a serving of commonly consumed fruit with a serving of berries per day on calories and nutrients in the 2000 calorie USDA dietary pattern healthy Mediterranean-Style.^a^

	2000 kcal Healthy Mediterranean-Style	2000 kcal Healthy Mediterranean-Style with berry replacement
Macronutrients		
Calories (kcal)	2029.42	2013.62
Protein (g)	108.85	109.44
Carbohydrates (g)	262.35	256.67
Fiber, total (g)	34.79	36.41
Soluble fiber, total (g)	3.23	3.40
Fat (g)	66.94	67.31
Fatty acids, total saturated (g)	16.01	16.00
Fatty acids, total monounsaturated (g)	26.76	26.82
Fatty acids, total polyunsaturated (g)	17.95	18.18
Trans fatty acids (g)	0.17	0.17
Cholesterol (mg)	310.30	310.30
Vitamins		
Vitamin A – RAE (mcg)	979.27	979.66
Vitamin B1 – Thiamin (mg)	1.56	1.55
Vitamin B2 – Riboflavin (mg)	2.59	2.58
Vitamin B3 – Niacin (mg)	23.52	24.03
Vitamin B6 (mg)	2.64	2.62
Vitamin B12 (mcg)	5.39	5.39
Biotin (mcg)	33.18	32.30
Vitamin C (mg)	150.78	171.81*
Vitamin D – IU (IU)	353.03	353.03
Vitamin E (alpha-tocopherol) (mg)	12.07	12.71
Folate (mcg)	519.03	533.82
Vitamin K (mcg)	273.39	286.35
Minerals		
Calcium (mg)	1107.85	1115.95
Copper (mg)	1.94	1.95
Iodine (mcg)	210.97	210.96
Iron (mg)	18.15	18.40
Magnesium (mg)	469.17	476.05
Phosphorus (mg)	1998.04	2001.65
Potassium (mg)	4057.54	4007.43
Selenium (mcg)	136.11	136.35
Sodium (mg)	1983.87	1983.33
Zinc (mg)	12.63	12.88
Anthocyanins (mg)	11.62	183.03*
Food cost ($)	72.96	79.68

^a^The 7 day berry replacement menu included 2 servings of fruit per day for a 2,000-calorie level with 7 servings of berries per week and 7 servings of commonly consumed fruit (apples, bananas, grapes) versus 14 servings of commonly consumed fruit in the USDA Dietary Pattern Healthy Mediterranean-Style. * > 10% increase or decrease from the USDA Dietary Pattern Healthy Mediterranean-Style.

Replacing a serving of commonly consumed fruit, such as apples, bananas, and grapes, with a serving of berries per day in the 2000 calorie HV USDA Dietary Patterns resulted in anthocyanins increasing by 93.8%. There was less than a 3.0% increase or decrease in calories, protein, total dietary fiber, fatty acids, and cholesterol. There was a 4.6% decrease in carbohydrates and a 10.2% increase in soluble fiber. Total sugar decreased by 9.2%. For vitamins there was an 11.9% increase in vitamin C, a 5.4% increase in vitamin E, a 5.1% increase in vitamin K, and less than a 3% increase or decrease in other vitamins. For minerals there was a 3.8% decrease in potassium, and less than or equal to a 3.0% decrease or increase in other minerals. Cost differential was an increase of $6.97 per week, which is a 10.5% increase (Table 4).

**Table 4.** Effect of replacing a serving of commonly consumed fruit with a serving of berries per day on calories and nutrients in the 2000 calorie USDA dietary pattern healthy Vegetarian.^a^

	2000 kcal Healthy Vegetarian	2000 kcal Healthy Vegetarian with berry replacement
Macronutrients		
Calories (kcal)	2008.14	1980.95
Protein (g)	91.64	94.78
Carbohydrates (g)	284.28	271.13
Fiber, total (g)	40.51	40.93
Soluble fiber, total (g)	2.81	3.10*
Fat (g)	63.67	64.86
Fatty acids, total saturated (g)	15.52	15.71
Fatty acids, total monounsaturated (g)	25.22	25.59
Fatty acids, total polyunsaturated (g)	17.49	17.94
Trans fatty acids (g)	0.16	0.16
Cholesterol (mg)	155.57	158.10
Vitamins		
Vitamin A – RAE (mcg)	1011.61	1013.94
Vitamin B1 – Thiamin (mg)	1.61	1.59
Vitamin B2 – Riboflavin (mg)	2.67	2.64
Vitamin B3 – Niacin (mg)	15.19	16.02
Vitamin B6 (mg)	2.15	2.09
Vitamin B12 (mcg)	4.50	4.60
Vitamin C (mg)	162.87	182.22*
Vitamin D – IU (IU)	289.55	290.44
Vitamin E (alpha-tocopherol) (mg)	11.24	11.84
Folate (mcg)	575.80	574.53
Folate, DFE (mcg DFE)	616.96	615.69
Vitamin K (mcg)	262.09	275.53
Minerals		
Calcium (mg)	1384.72	1387.56
Copper (mg)	2.10	2.08
Iodine (mcg)	235.90	236.31
Iron (mg)	19.95	19.87
Magnesium (mg)	492.31	489.24
Phosphorus (mg)	2002.22	2017.86
Potassium (mg)	4247.41	4084.06
Selenium (mcg)	95.42	96.17
Sodium (mg)	2328.99	2268.31
Zinc (mg)	12.17	12.56
Anthocyanins (mg)	11.51	185.85*
Food cost ($)	66.20	73.17

^a^The 7 day berry replacement menu included 2 servings of fruit per day for a 2,000-calorie level with 7 servings of berries per week and 7 servings of commonly consumed fruit (apples, bananas, grapes) versus 14 servings of commonly consumed fruit in the USDA Dietary Pattern Healthy Vegetarian. * > 10% increase or decrease from the USDA Dietary Pattern Healthy Vegetarian.

## Discussion

Results of this food pattern modeling analysis demonstrate that replacing a serving of commonly consumed fruit, such as apples, bananas, and grapes, with a serving of berries, such as blueberries, blackberries, raspberries, and strawberries, yields favorable effects on the nutrient and dietary component profiles of the USDA Dietary Patterns. Most notably, the increase in anthocyanins is the most significant finding. This analysis methodically quantifies the striking improvement in anthocyanin intake achievable through the simple inclusion of just one daily serving of berries within USDA Dietary Patterns. Importantly, these benefits were achieved without increases in calories, sodium, saturated fat, or cholesterol across the 2000 calorie per day HUS, HMS, and HV USDA Dietary Patterns.

Anthocyanins increased by 93.8% in HUS, HMS, and HV USDA Dietary Patterns. Berries are dietary sources of polyphenols, specifically anthocyanins. The US Department of Health & Human Services, National Institutes of Health, Office of Dietary Supplements has proposed the term bioactives or bioactive food compounds for use in referring to constituents in foods or dietary supplements other than those needed to meet basic human nutritional needs yet are responsible for changes in health status. Anthocyanins would be considered a bioactive food compound per this definition [12]. Regular consumption of anthocyanins, especially food sources such as berries, have been associated with potential health benefits including decreased risk of overall mortality, cardiovascular disease, type 2 diabetes, and improved cognitive function [13–16]. Additionally, there have been improvements in cardiometabolic risk factors such as blood concentrations of total cholesterol, lipoproteins, and inflammatory biomarkers, as well as improved vasodilation, blood flow, and elasticity of blood vessels [4,17,18]. There is no guideline recommendation for food-based intake of anthocyanins, however there is a framework for developing recommended intakes of bioactive food compounds [19]. Current intake of anthocyanins is 13.8 mg/day for adult males age > 20 years and 16.4 mg/day for adult females age > 20 years as determined by NHANES 2017–2018 data [20]. Interestingly, there are recently published 2023 Nordic Nutrition Recommendations, which highlight the importance of including berries in the diet for health benefits [21]. The Nordic Diet recommends consuming a daily intake of 50 to 100 g of berries [22].

Dietary fiber is considered a dietary component of public health concern in the 2020–2025 Dietary Guidelines for Americans [2]. Dietary fiber increased by 4.7% and 5.1% in the food pattern modeling analysis for the HUS and HMS USDA Dietary Patterns, respectively, with minimal increases (<1.5%) in the HV USDA Dietary Patterns. Soluble fiber, however, increased by 10.1%, 5.3%, and 10.2% in the HUS, HMS, and the HV USDA Dietary Patterns, respectively. Dietary fiber is known to improve the health of the gastrointestinal tract along with playing a crucial role in the prevention and management of cardiovascular disease, diabetes mellitus, and others [23]. The Food and Drug Administration (FDA) has approved several fiber-related health claims which include soluble fiber. The health claims emphasize increased consumption of fiber rich foods such as fruits, vegetables, and whole grains [24]. The dietary fiber DRIs Adequate Intake (AI) for adult males and females, 19 to 50 years of age, are 38 g and 25 g/day, respectively [25]. However, research shows that there is inadequate dietary fiber intake across all US population groups based on intake estimates from What We Eat in America (WWEIA)/National Health and Nutrition Examination Survey (NHANES) 2017–2019 data. Current intake of dietary fiber is 18.4 g/day for adult males age > 20 years and 15.5 g/day for adult females age > 20 years as determined by NHANES 2017–2018 data [20].

Amounts of vitamin C increased with replacing commonly consumed fruit, such as apples, bananas, and grapes, with a serving of berries per day to the 2000 calorie HUS, HMS, and HV USDA Dietary Patterns. Vitamin C increased by 11.9% to 16.0% in the food pattern modeling analysis. Vitamin C, a water-soluble vitamin, is an essential micronutrient which functions as an antioxidant and as a co-substrate for enzyme activity [26]. The Recommended Dietary Allowance (RDA) of vitamin C for adult males and females, > 19 years of age, are 90 mg and 75 mg/day, respectively [27]. However, research shows that there is inadequate vitamin C intake across all US population groups based on intake estimates from WWEIA/NHANES 2017–2019 data. Current intake of vitamin C is 80.4 mg/day for adult males age > 20 years and 72.5 mg/day for adult females age > 20 years as determined by NHANES 2017–2018 data [20].

Other vitamins that were increased by replacing commonly consumed fruit, such as apples, bananas, and grapes, with a serving of berries in the Dietary Patterns were vitamin E and vitamin K in the food pattern modeling analysis. Vitamin E was increased by 5.3% to 6.0% for the HUS, HMS, and the HV USDA Dietary Patterns. Vitamin E’s principal function is as an antioxidant [28]. The RDA for vitamin E for adult males and females > 19 years of age, is 15 mg of naturally occurring alpha-tocopherol/day [27]. Current intake of vitamin E (alpha-tocopherol) is 10.5 mg/day for adult males age > 20 years and 8.6 mg/day for adult females age > 20 years as determined by NHANES 2017–2018 data [20]. These intake estimates may be low due to underestimated cooking oil consumption. Vitamin E is found primarily in plant foods such as nuts, seeds, and the oils of plants. Interestingly, berry seeds may be an unexplored source of vitamin E [3,28]. Vitamin K increased by 4.7% to 5.4% for the HUS, HMS, and the HV USDA Dietary Patterns. Vitamin K is the generic name for a family of compounds with a common chemical structure of 2-methyl-1,4-naphthoquinone. It is known as an essential factor in blood coagulation with some evidence showing beneficial effects on bone and cardiovascular health [29]. The vitamin K DRIs AI for adult males and females, > 19 years of age are 120 µg and 90 µg, respectively [30]. Current intake of vitamin K is 126.9 µg/day for adult males age > 20 years and 128.6 µg/day for adult females age > 20 years as determined by NHANES 2017–2018 data [20]. Dietary vitamin K, provided generally as phylloquinone, is found primarily in plant foods such as leafy green vegetables. Berries such as blackberries (14.7–25.1 µg per 100 g) and blueberries (14.7–27.2 μg per 100 g) contain vitamin K. Oils from plants are also a major source of vitamin K and may be more bioavailable versus plant foods [31,32].

The food cost of replacing a serving of commonly consumed fruit, such as apples, bananas, and grapes, with a serving of berries per day had an increase of approximately 10% across the 3 USDA Dietary Patterns. The USDA Cost of Food at Home US Average for 2024 shows that moderate-cost weekly food plans are $87.60 and $73.90 for males and females 19 to 50 years of age, respectively [33]. The weekly food costs for the 7-day berry replacement menus ranged from a low of $73.17 for the HV USDA Dietary Pattern to a high of $79.68 for the HMS USDA Dietary Patterns. These costs were similar to those in the USDA’s moderate-cost weekly food plan Cost of Food at Home for 2024.

Fruit provides a heterogeneous array of nutrients and bioactive compounds, including polyphenols, that vary according to botanical classification, pigmentation, and morphological characteristics, suggesting that a more refined subcategorization of the fruit group within dietary guidance may be scientifically justified. For example, these subgroups may include berries (e.g. blackberries, blueberries, raspberries, strawberries), citrus fruits (e.g. oranges, grapefruits, lemons), pome fruits (e.g. apples and pears), stone fruits (e.g. cherries, peaches, plums), tropical fruits (e.g. bananas, mangoes, pineapples) and others. The development of fruit subgroups that reflect compositional and functional differences may promote increased variety in fruit consumption and improve overall dietary quality [7]. Incorporating more than two fruit subgroups into dietary recommendations, especially on a weekly basis, may encourage greater diversity in fruit intake and improve the unique health-promoting properties of different fruit types. In addition, the vegetable group in the DGA is a weekly recommendation. However, this approach raises important considerations around feasibility, including consumer education and food access.

A key strength of the study was the use of the USDA’s food pattern modeling approach to create the three 7-day replacement menus. These menus were designed to reflect the substitution of berries for commonly consumed fruits in the HUS, HMS, and HV USDA Dietary Pattern models at the 2000-calorie level. The primary limitation of the study was that the results demonstrated the maximum potential effects on nutrients and dietary components based on the food modeling approach, which may not accurately represent actual individual food intake.

## Conclusion

Food pattern modeling analysis provides insights into the nutritional benefits of replacing one serving of commonly consumed fruit with one serving of berries per day in the USDA Dietary Patterns at the 2000-calorie level. The addition of berries to the USDA Dietary Patterns increased beneficial dietary components and nutrients with minimal change in calories, macronutrients, fatty acids, and cholesterol. Food costs increased by approximately 10%, but they remained comparable to those outlined in the USDA’s moderate-cost weekly food plan, Cost of Food at Home for 2024.

## Supplementary Material

Appendix_1 - Clean.docx

## Data Availability

Data are available from the corresponding author upon reasonable request.
