# Supplementary material for: Effect of replacing commonly consumed fruit in the United States with berries in the USDA healthy Dietary Patterns: a modeling analysis
Source: Ann Med. 2025 Jun 12;57(1):2517817. doi: 10.1080/07853890.2025.2517817 (PMC12164377; doi:10.1080/07853890.2025.2517817)
Supplement: Appendix_1 - Clean.docx [file IANN_A_2517817_SM1364.docx]

| **HEALTHY U.S. PATTERN** | **DAY 1 + Swap** | **DAY 2 + Swap** | **DAY 3 + Swap** | **DAY 4 + Swap** |
| --- | --- | --- | --- | --- |
| **Breakfast** | **Toast with peanut butter:**   - 1 slice (1 oz) whole wheat bread - 1 tbsp peanut butter   **Fruit:**   - **1 small apple, sliced**   **Milk:**   - 8 oz nonfat milk, with vitamins A & D | **Scrambled eggs with a side of hashbrowns and sliced tomato:**   - 2 eggs - 2 oz hash browns - 1 tsp olive oil - 1 large tomato, sliced   **Coffee:**   - 8 oz coffee - 1 oz nonfat milk, with vitamins A & D | **Yogurt topped with cereal and fruit:**   - 1 cup nonfat Greek yogurt - 1 cup corn flakes cereal - **1 large banana / 1 cup frozen mixed berries**   **Coffee:**   - 8 oz coffee - 1 oz nonfat milk, with vitamins A & D | **Cereal**   - ¾ cup corn flakes - 8 oz nonfat milk, with vitamins A & D   **Fruit:**   - **1 cup green grapes**   **Hard Boiled Egg:**   - 1 hardboiled egg   **Coffee:**   - 8 oz coffee |
| **Morning Snack** | **Yogurt topped with fruit and corn flakes:**   - 1 cup nonfat Greek yogurt - 1 cup corn flakes - **1 large banana, sliced / 1 cup blueberries** - 1 tsp honey | **Cheese, crackers and fruit:**   - 1 stick (1 oz) part skim mozzarella string cheese - 1.5 oz whole wheat crackers - **1 cup green grapes** | **Cheese, crackers and fruit:**   - 1 oz cheddar cheese, reduced fat - 1.75 oz whole wheat crackers - **1 small apple, sliced** | **Cottage cheese topped with sliced cucumber and sunflower seeds:**   - 1 cup low fat cottage cheese - 1 cup cucumber, sliced - 0.5 oz sunflower seeds |
| **Lunch** | **Chopped spinach salad with chicken, side of pita:**   - 1 cup chopped spinach - 2 oz chicken breast, roasted with 0.5 tbsp canola oil - ½ cup whole cherry tomatoes - 0.5 oz cheddar cheese, shredded - 0.5 oz sunflower seeds - 1 tbsp Ranch dressing - 1 large whole wheat pita   **Iced Tea:**   - 8 oz unsweetened iced tea | **Turkey sandwich:**   - 2 slices (2 oz) whole wheat bread - 1 oz turkey, deli cut - 1 thin slice reduced fat American cheese - 1 tsp mustard - 1 medium leaf iceberg lettuce   **Apple:**   - **1 small apple / 1 cup fresh mixed berries**   **Milk:**   - 8 oz nonfat milk fortified w/ Vitamins A & D | **Southwest chicken salad with tortilla chips:**   - 1 ¼ cup mixed greens - 2 oz chicken breast, roasted - ½ cup quinoa, cooked - 1 cup whole cherry tomatoes - ¼ cup corn, boiled from frozen - 0.25 oz sunflower seeds - 1 tbsp Ranch dressing - 2 oz tortilla chips   **Iced Tea:**  8 oz unsweetened iced tea | **Black bean & rice bowl:**   - ½ cup white rice, cooked - ½ cup black beans, canned, low sodium - ½ red bell pepper - ½ green bell pepper - ¼ cup white onion, chopped - 1 tbsp olive oil   **Milk:**   - 8 oz nonfat milk fortified w/ Vitamins A & D |
| **Afternoon Snack** | **Crackers and deli turkey with almonds:**   - 1.5 oz saltine crackers - 0.5 oz turkey, deli cut - 8 whole fresh almonds, roasted, unsalted | **Homemade Trail Mix:**   - 1.25 oz dark chocolate - 0.25 oz walnuts, salted, dry roasted - 3 cups air popped popcorn | **Popcorn:**   - 3 cups air popped popcorn | **Hummus with sliced carrots and pretzels:**   - ½ cup hummus - 1 medium carrot, sliced - 10 pretzels   **Fruit:**   - **1 small apple / 1 cup blueberries** |
| **Dinner** | **Steak, baked potato and broccoli:**   - 3 oz broiled lean top sirloin - 5 oz baked russet potato, skin on (medium potato) - 1 tsp sour cream - 1 cup boiled broccoli   **Milk:**   - 8 oz nonfat milk fortified w/ Vitamins A & D | **Balsamic chicken and pasta with spinach and asparagus:**   - 3 oz chicken breast, roasted - 1 cup whole wheat pasta, spaghetti - 2 cups chopped spinach - ½ cup asparagus, unsalted, canned - 1 tbsp olive oil - 1 tbsp balsamic vinegar   **Milk:**   - 8 oz nonfat milk fortified w/ Vitamins A & D | **Baked tilapia, wild rice and lentils mix and veggies:**   - 2 oz tilapia, baked - 1 tbsp olive oil - 1 tsp lemon juice - ½ cup wild rice, cooked - ¼ cup lentils, boiled - 1 cup broccoli - 0.5 oz parmesan cheese, grated   **Milk:**   - 8 oz nonfat milk fortified w/ Vitamins A & D | **Tofu noodle stir-fry:**   - 3 oz firm tofu - ¾ cup soba noodles, cooked - ½ cup carrots, chopped - 1/3 cup mushrooms, sliced - 1 garlic clove - 1 tbsp sesame oil - 1 tbsp ginger root   **Milk:**   - 8 oz nonfat milk, with vitamins A & D |
| **[Menu Footnote]** | *This menu item follows the Healthy US dietary pattern and features a variety of whole grain and refined grains* | *This menu item follows the Healthy US dietary pattern and features a variety of whole grain and refined grains* | *This menu item follows the Healthy US dietary pattern and features a variety of whole grain and refined grains* | *This menu item follows the Healthy US dietary pattern and features a variety of whole grain and refined grains* |

|  | **DAY 5 + Swap** | **DAY 6 + Swap** | **Day 7 + Swap** |  |
| --- | --- | --- | --- | --- |
| **Breakfast** | **Fried egg with sliced tomatoes and sauteed spinach:**   - 1 large egg - 0.5 tbsp olive oil - 1 medium tomato, sliced - 0.5 cup spinach, fresh, chopped   **Hash browns:**   - 2 oz hash browns   **Milk:**   - 8 oz nonfat milk fortified w/ Vitamins A & D | **Oatmeal prepared with milk:**   - 1 cup oatmeal, cooked - 4 oz nonfat milk fortified w/ Vitamins A&D   **Hardboiled egg:**   - 1 large egg   **Fruit:**   - **1 cup green grapes**   **Coffee:**   - 8 oz coffee | **Spinach and feta omelet:**   - 1 large egg - 1 oz feta cheese - 0.5 cup fresh spinach - 0.5 tbsp olive oil   **Toast:**   - 1 oz sourdough bread   **Fruit:**   - **1 large banana**   **Milk:**   - 8 oz nonfat milk fortified w/ Vitamins A & D |  |
| **Morning Snack** | **Banana with peanut butter and sunflower seeds:**   - 1 tbsp peanut butter - 0.25 oz sunflower seeds   **Fruit:**   - **1 large banana** | **Turkey & cheese rollups, fruit and popcorn:**   - 1 oz turkey, deli cut - 0.75 oz low fat cheddar cheese - 3 cups air popped popcorn - **1 small apple / 1 cup fresh mixed berries** | **Hummus with carrot slices and pretzels:**   - ¼ cup hummus - 1 medium carrot, sliced - 1 oz pretzels |  |
| **Lunch** | **Tuna pita sandwich with a side of carrot sticks and ranch dressing:**   - 1 large whole wheat pita - 1 can tuna, canned in water, drained - 1.5 tsp light mayo - ¼ cup celery, diced - 1 slice Swiss cheese - 1 large carrot, sliced - 1 tbsp fat-free ranch dressing   **Iced Tea:**  8 oz unsweetened iced tea | **Chicken & rice bowl with side of tortilla chips:**   - ½ cup brown rice, cooked - 3 oz ground chicken, cooked - 1 tbsp canola oil - ¼ cup tomato, chopped - ¼ cup white onion, chopped - ¼ cup green pepper, chopped - ½ cup black beans, canned, low sodium - 1 oz tortilla chips   **Milk:**   - 8 oz nonfat milk fortified w/ Vitamins A & D | **Grilled chicken pasta salad:**   - ¾ cup cooked pasta, rotini - 2 oz chicken, grilled - ½ cup broccoli, chopped - ½ cup red onion, chopped - 0.75 tbsp olive oil - 1 tbsp red wine vinegar - 1 tbsp basil leaves, chopped   **Fruit:**   - **1 cup green grapes / 1 cup blueberries**   **Iced Tea:**  8 oz unsweetened iced tea |  |
| **Afternoon Snack** | **Yogurt with cereal and granola:**   - ¾ cup nonfat Greek yogurt - ½ cup corn flakes cereal - 2 tbsp granola   **Chocolate:**   - 0.25 oz dark chocolate   **Fruit:**   - **1 cup green grapes / 1 cup frozen blueberries** | **Veggies and crackers with hummus:**   - 3 stalks celery - ¼ cup hummus - 3 whole wheat crackers | **Popcorn and dark chocolate:**   - 1 cup air popped popcorn - 1 oz dark chocolate |  |
| **Dinner** | **Chicken thighs with naan flatbread and roasted vegetables:**   - 2 oz chicken thighs, skinless, roasted - 1 piece whole wheat naan - 4 tbsp hummus - ½ cup green peppers, chopped - ½ cup zucchini, boiled, sliced - 0.5 tbsp olive oil   **Milk:**   - 8 oz nonfat milk fortified w/ Vitamins A & D | **Salmon over quinoa and veggies/greens:**   - 2 oz salmon fillet, cooked - 1 cup quinoa, cooked - 1.5 cups chopped spinach - 0.5 oz feta cheese - ¼ cup cucumber, sliced - 2 medium asparagus spears, chopped   **Milk:**   - 8 oz nonfat milk fortified w/ Vitamins A & D | **Turkey & black bean chili with a side of cornbread:**   - 1.5 oz ground turkey - ½ cup black beans, canned, low sodium - ¼ cup white onion, diced - 6 oz (~1/2 can) crushed canned tomatoes - ½ cup red bell pepper, chopped - 1 piece cornbread   **Milk:**   - 8 oz nonfat milk fortified w/ Vitamins A & D |  |
| **[Menu Footnote]** | *This menu item follows the Healthy US dietary pattern and features a variety of whole grain and refined grains* | *This menu item follows the Healthy US dietary pattern and features a variety of whole grain and refined grains* | *This menu item follows the Healthy US dietary pattern and features a variety of whole grain and refined grains* |  |

| **VEGETARIAN** | **DAY 1 + Swap** | **DAY 2 + Swap** | **DAY 3 + Swap** | **DAY 4 + Swap** |
| --- | --- | --- | --- | --- |
| **Breakfast** | **Toast with peanut butter:**   - 1 slice (1 oz) whole wheat bread - 1 tbsp peanut butter   **Fruit:**   - **1 small apple, sliced**   **Milk:**   - 8 oz nonfat milk, with vitamins A & D | **Scrambled eggs with a side of hashbrowns and sliced tomato:**   - 1 egg - 2 oz hash browns - 1 tsp olive oil - 1 large tomato, sliced   **Coffee:**   - 8 oz coffee - 1 oz nonfat milk, with vitamins A & D | **Yogurt topped with cereal and fruit:**   - 1 cup nonfat Greek yogurt - 1 cup corn flakes cereal - **1 large banana / 1 cup frozen mixed berries**   **Coffee:**   - 8 oz coffee - 1 oz nonfat milk, with vitamins A & D | **Cereal**   - ¾ cup corn flakes - 8 oz nonfat milk, with vitamins A & D   **Fruit:**   - **1 cup green grapes**   **Hard Boiled Egg:**   - 1 hardboiled egg   **Coffee:**   - 8 oz coffee |
| **Morning Snack** | **Yogurt topped with fruit and corn flakes:**   - 1 cup nonfat Greek yogurt - 1 cup corn flakes - **1 large banana, sliced / 1 cup blueberries** - 1 tsp honey | **Cheese, crackers and fruit:**   - 1 stick (1 oz) part skim mozzarella string cheese - 1.5 oz whole wheat crackers - **1 cup green grapes** | **Cheese, crackers and fruit:**   - 1 oz cheddar cheese, reduced fat - 1.75 oz whole wheat crackers - **1 small apple, sliced** | **Cottage cheese topped with sliced cucumber and sunflower seeds:**   - 1 cup low fat cottage cheese - 1 cup cucumber, sliced - 0.5 oz sunflower seeds |
| **Lunch** | **Chopped spinach salad with chili, side of pita:**   - 1 cup chopped spinach - ½ c. vegetarian chili beans - ½ cup whole cherry tomatoes - 0.5 oz cheddar cheese, shredded - 0.5 oz sunflower seeds - 1 tbsp Ranch dressing - 1 large whole wheat pita   **Iced Tea:**   - 8 oz unsweetened iced tea | **Sandwich:**   - 2 slices (2 oz) whole wheat bread - 2 thin slices reduced fat American cheese - 1 tsp mustard - *1 c. tomato soup*   **Apple:**   - **1 small apple / 1 cup fresh mixed berries**   **Milk:**   - 8 oz nonfat milk fortified w/ Vitamins A & D | **Southwest salad with tortilla chips:**   - 1 ¼ cup mixed greens - 2 oz edamame - ½ cup quinoa, cooked - 1 cup whole cherry tomatoes - ¼ cup corn, boiled from frozen - 0.25 oz sunflower seeds - 1 tbsp Ranch dressing - 2 oz tortilla chips   **Iced Tea:**  8 oz unsweetened iced tea | **Black bean & rice bowl:**   - ½ cup white rice, cooked - ½ cup black beans, canned, low sodium - ½ red bell pepper - ½ green bell pepper - ¼ cup white onion, chopped - 1 tbsp olive oil   **Milk:**   - 8 oz nonfat milk fortified w/ Vitamins A & D |
| **Afternoon Snack** | **Crackers and cheese with almonds:**   - 1.5 oz saltine crackers - 1 oz smoked Gouda - 8 whole fresh almonds, roasted, unsalted | **Homemade Trail Mix:**   - 1.25 oz dark chocolate - 0.25 oz walnuts, salted, dry roasted - 3 cups air popped popcorn | **Popcorn:**   - 3 cups air popped popcorn | **Hummus with sliced carrots and pretzels:**   - ½ cup hummus - 1 medium carrot, sliced - 10 pretzels   **Fruit:**   - **1 small apple / 1 cup blueberries** |
| **Dinner** | **Tofu, baked potato and broccoli:**   - 3 oz. Fried Tofu stir fried with ½ c carrots, ½ c broccoli - .5 c brown rice - .5 Tbsp canola oil - 2 tsp. soy sauce   **Milk:**   - 8 oz nonfat milk fortified w/ Vitamins A & D | **Balsamic pasta with spinach, edamame and asparagus:**   - 2 oz. edamame - 1 cup whole wheat pasta, spaghetti - 2 cups chopped spinach - ½ cup asparagus, unsalted, canned - 1 tbsp olive oil - 1 tbsp balsamic vinegar   **Milk:**   - 8 oz nonfat milk fortified w/ Vitamins A & D | **Wild rice, beans and lentils mix and veggies:**   - ¼ c. black beans - 1 tbsp olive oil - ½ cup wild rice, cooked - ¼ cup lentils, boiled - 1 cup broccoli - 0.5 oz parmesan cheese, grated   **Milk:**   - 8 oz nonfat milk fortified w/ Vitamins A & D | **Tofu noodle stir-fry:**   - 3 oz firm tofu - ¾ cup soba noodles, cooked - ½ cup carrots, chopped - 1/3 cup mushrooms, sliced - 1 garlic clove - 1 tbsp sesame oil - 1 tbsp ginger root - ½ c brown rice   **Milk:**   - 8 oz nonfat milk, with vitamins A & D |
| **[Menu Footnote]** | *This menu item follows the Healthy US dietary pattern and features a variety of whole grain and refined grains* | *This menu item follows the Healthy US dietary pattern and features a variety of whole grain and refined grains* | *This menu item follows the Healthy US dietary pattern and features a variety of whole grain and refined grains* | *This menu item follows the Healthy US dietary pattern and features a variety of whole grain and refined grains* |

|  | **DAY 5 + Swap** | **DAY 6 + Swap** | **Day 7 + Swap** |  |
| --- | --- | --- | --- | --- |
| **Breakfast** | **Fried egg with sliced tomatoes and sauteed spinach:**   - 1 large egg - 0.5 tbsp olive oil - 1 medium tomato, sliced - 0.5 cup spinach, fresh, chopped   **Hash browns:**   - 2 oz hash browns   **Milk:**   - 8 oz nonfat milk fortified w/ Vitamins A & D | **Oatmeal prepared with milk:**   - 1 cup oatmeal, cooked - 4 oz nonfat milk fortified w/ Vitamins A&D - 1 Tbsp peanut butter   **Fruit:**   - **1 cup green grapes**   **Coffee:**   - 8 oz coffee | **Spinach and feta omelet:**   - 1 large egg - 1 oz feta cheese - 0.5 cup fresh spinach - 0.5 tbsp olive oil   **Toast:**   - 1 oz sourdough bread   **Fruit:**   - **1 large banana**   **Milk:**   - 8 oz nonfat milk fortified w/ Vitamins A & D |  |
| **Morning Snack** | **Banana with peanut butter and sunflower seeds:**   - 1 tbsp peanut butter - 0.25 oz sunflower seeds   **Fruit:**   - **1 large banana** | **Cheese, fruit and popcorn:**   - 1.5 oz low fat cheddar cheese - 3 cups air popped popcorn - **1 small apple / 1 cup fresh mixed berries** | **Hummus with carrot slices and pretzels:**   - ¼ cup hummus - 1 medium carrot, sliced - 1 oz pretzels |  |
| **Lunch** | **Hummus & pita with a side of carrot sticks and ranch dressing:**   - 1 large whole wheat pita, cut into triangles - ½ c hummus - ¼ cup celery, diced - 1 slice Swiss cheese - 1 large carrot, sliced - 1 tbsp fat-free ranch dressing   **Iced Tea:**  8 oz unsweetened iced tea | **Bean & rice bowl with side of tortilla chips:**   - ½ cup brown rice, cooked - 1 tbsp canola oil - ¼ cup tomato, chopped - ¼ cup white onion, chopped - ¼ cup green pepper, chopped - 1 cup black beans, canned, low sodium - 1 oz tortilla chips - 2 oz shredded cheese   **Milk:**   - 8 oz nonfat milk fortified w/ Vitamins A & D | **Pasta salad:**   - ¾ cup cooked pasta, rotini - ½ c 3-bean salad - ½ cup broccoli, chopped - ½ cup red onion, chopped - 0.75 tbsp olive oil - 1 tbsp red wine vinegar - 1 tbsp basil leaves, chopped   **Fruit:**   - **1 cup green grapes / 1 cup blueberries**   **Iced Tea:**  8 oz unsweetened iced tea |  |
| **Afternoon Snack** | **Yogurt with cereal and granola:**   - ¾ cup nonfat Greek yogurt - ½ cup corn flakes cereal - 2 tbsp granola   **Chocolate:**   - 0.25 oz dark chocolate   **Fruit:**   - **1 cup green grapes / 1 cup frozen blueberries** | **Veggies and crackers with hummus:**   - 3 stalks celery - ¼ cup hummus - 3 whole wheat crackers | **Popcorn and dark chocolate:**   - 1 cup air popped popcorn - 1 oz dark chocolate |  |
| **Dinner** | **Naan flatbread and roasted vegetables:**   - 1 piece whole wheat naan - 2 oz tofu - ½ cup green peppers, chopped - ½ c. roasted yam - ½ cup zucchini, sliced, roasted - 0.5 tbsp olive oil   **Milk:**   - 8 oz nonfat milk fortified w/ Vitamins A & D | **Quinoa and veggies/greens salad**   - 1 cup quinoa, cooked - 1.5 cups chopped spinach - 0.5 oz feta cheese - ¼ cup cucumber, sliced - ¼ c tomato - 2 medium asparagus spears, chopped - 2 oz whole wheat pita   **Milk:**   - 8 oz nonfat milk fortified w/ Vitamins A & D | **Black bean chili with a side of cornbread:**   - 1 cup black beans, canned, low sodium - ¼ cup white onion, diced - 6 oz (~1/2 can) crushed canned tomatoes - ½ cup red bell pepper, chopped - 1 piece cornbread   **Milk:**   - 8 oz nonfat milk fortified w/ Vitamins A & D |  |
| **[Menu Footnote]** | *This menu item follows the Healthy US dietary pattern and features a variety of whole grain and refined grains* | *This menu item follows the Healthy US dietary pattern and features a variety of whole grain and refined grains* | *This menu item follows the Healthy US dietary pattern and features a variety of whole grain and refined grains* |  |

| **MEDITERRANEAN** | **DAY 1 + Swap** | **DAY 2 + Swap** | **DAY 3 + Swap** | **DAY 4 + Swap** |
| --- | --- | --- | --- | --- |
| **Breakfast** | **Toast with peanut butter:**   - 1 slice (1 oz) whole wheat bread - 1 tbsp peanut butter   **Fruit:**   - **1 medium apple, sliced**   **Milk:**   - 8 oz nonfat milk, with vitamins A & D | **Scrambled eggs with a side of hashbrowns and sliced tomato:**   - 2 eggs - 2 oz hash browns - 1 tsp olive oil - 1 large tomato, sliced - ½ orange   **Coffee:**   - 8 oz coffee - 1 oz nonfat milk, with vitamins A & D | **Yogurt topped with cereal and fruit:**   - 1 cup nonfat Greek yogurt - 1 cup corn flakes cereal - **1 large banana / 1 cup frozen mixed berries**   **Coffee:**   - 8 oz coffee - 1 oz nonfat milk, with vitamins A & D | **Cereal**   - ¾ cup corn flakes - 8 oz nonfat milk, with vitamins A & D   **Fruit:**   - **1 cup green grapes**   **Hard Boiled Egg:**   - 1 hardboiled egg   **Coffee:**   - 8 oz coffee |
| **Morning Snack** | **Yogurt topped with fruit and corn flakes:**   - 1 cup nonfat Greek yogurt - 1 cup corn flakes - **1 large banana, sliced / 1 cup blueberries** - 1 tsp honey | **Cheese, crackers and fruit:**   - 1 stick (1 oz) part skim mozzarella string cheese - 1.5 oz whole wheat crackers - **1*.5* cup green grapes** | **Cheese, crackers and fruit:**   - 1 oz cheddar cheese, reduced fat - 1.75 oz whole wheat crackers - **1 small apple, sliced** | **Cottage cheese topped with sliced cucumber and sunflower seeds:**   - 1 cup low fat cottage cheese - 1 cup cucumber, sliced - 0.5 oz sunflower seeds |
| **Lunch** | **Chopped spinach salad with shrimp, side of pita:**   - 1 cup chopped spinach - 2 oz shrimp, roasted with 0.5 tbsp canola oil - ½ cup whole cherry tomatoes - 0.5 oz cheddar cheese, shredded - 0.5 oz sunflower seeds - 1 tbsp Ranch dressing - 1 large whole wheat pita   **Iced Tea:**   - 8 oz unsweetened iced tea | **Tuna sandwich:**   - 2 slices (2 oz) whole wheat bread - 2 oz tuna - 1 Tbsp mayo - 1 tsp mustard - 1 medium leaf iceberg lettuce   **Apple:**   - **1 small apple / 1 cup fresh mixed berries**   **Milk:**   - 8 oz nonfat milk fortified w/ Vitamins A & D | **Southwest chicken salad with tortilla chips:**   - 1 ¼ cup mixed greens - 2 oz chicken breast, roasted - ½ cup quinoa, cooked - 1 cup whole cherry tomatoes - ¼ cup corn, boiled from frozen - 0.25 oz sunflower seeds - 1 tbsp Ranch dressing - 2 oz tortilla chips   **Iced Tea:**  8 oz unsweetened iced tea | **Black bean & rice bowl:**   - ½ cup white rice, cooked - ½ cup black beans, canned, low sodium - ½ red bell pepper - ½ green bell pepper - ¼ cup white onion, chopped - 1 tbsp olive oil - ½ orange |
| **Afternoon Snack** | **Crackers and deli turkey with almonds:**   - 1.5 oz saltine crackers - 0.5 oz turkey, deli cut - 8 whole fresh almonds, roasted, unsalted | **Homemade Trail Mix:**   - 1.25 oz dark chocolate - 0.25 oz walnuts, salted, dry roasted - 3 cups air popped popcorn | **Popcorn:**   - 3 cups air popped popcorn | **Hummus with sliced carrots and pretzels:**   - ½ cup hummus - 1 medium carrot, sliced - 10 pretzels   **Fruit:**   - **1 small apple / 1 cup blueberries** |
| **Dinner** | **Steak, baked potato and broccoli:**   - 3 oz broiled lean top sirloin - 5 oz baked russet potato, skin on (medium potato) - 1 tsp sour cream - 1 cup boiled broccoli | **Balsamic chicken and pasta with spinach and asparagus:**   - 3 oz chicken breast, roasted - 1 cup whole wheat pasta, spaghetti - 2 cups chopped spinach - ½ cup asparagus, unsalted, canned - 1 tbsp olive oil - 1 tbsp balsamic vinegar | **Baked tilapia, wild rice and lentils mix and veggies:**   - 2 oz tilapia, baked - 1 tbsp olive oil - 1 tsp lemon juice - ½ cup wild rice, cooked - ¼ cup lentils, boiled - 1 cup broccoli - 0.5 oz parmesan cheese, grated - 1 small orange   **Milk:**   - 8 oz nonfat milk fortified w/ Vitamins A & D | **Tofu noodle stir-fry:**   - 3 oz cod - ¾ cup soba noodles, cooked - ½ cup carrots, chopped - 1/3 cup mushrooms, sliced - 1 garlic clove - 1 tbsp sesame oil - 1 tbsp ginger root   **Milk:**   - 8 oz nonfat milk, with vitamins A & D |
| **[Menu Footnote]** | *This menu item follows the Healthy US dietary pattern and features a variety of whole grain and refined grains* | *This menu item follows the Healthy US dietary pattern and features a variety of whole grain and refined grains* | *This menu item follows the Healthy US dietary pattern and features a variety of whole grain and refined grains* | *This menu item follows the Healthy US dietary pattern and features a variety of whole grain and refined grains* |
|  |  |  |  |  |
|  | **DAY 5 + Swap** | **DAY 6 + Swap** | **Day 7 + Swap** |  |
| **Breakfast** | **Fried egg with sliced tomatoes and sauteed spinach:**   - 1 large egg - 0.5 tbsp olive oil - 1 medium tomato, sliced - 0.5 cup spinach, fresh, chopped   **Hash browns:**   - 2 oz hash browns   **Milk:**   - 4 oz nonfat milk fortified w/ Vitamins A & D - 4 oz. orange juice | **Oatmeal prepared with milk:**   - 1 cup oatmeal, cooked - 4 oz nonfat milk fortified w/ Vitamins A&D - *1 small apple*   **Hardboiled egg:**   - 1 large egg   **Fruit:**   - **1 cup green grapes**   **Coffee:**   - 8 oz coffee | **Spinach and feta omelet:**   - 1 large egg - 1 oz feta cheese - 0.5 cup fresh spinach - 0.5 tbsp olive oil   **Toast:**   - 1 oz sourdough bread   **Fruit:**   - **1 large banana**   **Milk:**   - 8 oz nonfat milk fortified w/ Vitamins A & D |  |
| **Morning Snack** | **Banana with peanut butter and sunflower seeds:**   - 1 tbsp peanut butter - 0.25 oz sunflower seeds   **Fruit:**   - **1 large banana** | **Turkey & cheese rollups, fruit and popcorn:**   - 1 oz turkey, deli cut - 0.75 oz low fat cheddar cheese - 3 cups air popped popcorn - **1 small apple / 1 cup fresh mixed berries** | **Hummus with carrot slices and pretzels:**   - ¼ cup hummus - 1 medium carrot, sliced - 1 oz pretzels |  |
| **Lunch** | **Tuna pita sandwich with a side of carrot sticks and ranch dressing:**   - 1 large whole wheat pita - 1 can tuna, canned in water, drained - 1.5 tsp light mayo - ¼ cup celery, diced - 1 slice Swiss cheese - 1 large carrot, sliced - 1 tbsp fat-free ranch dressing   **Iced Tea:**  8 oz unsweetened iced tea | **Chicken & rice bowl with side of tortilla chips:**   - ½ cup brown rice, cooked - 3 oz ground chicken, cooked - 1 tbsp canola oil - ¼ cup tomato, chopped - ¼ cup white onion, chopped - ¼ cup green pepper, chopped - ½ cup black beans, canned, low sodium - 1 oz tortilla chips   **Milk:**   - 8 oz nonfat milk fortified w/ Vitamins A & D | **Grilled prawn pasta salad:**   - ¾ cup cooked pasta, rotini - 2 oz prawns grilled - ½ cup broccoli, chopped - ½ cup red onion, chopped - 0.75 tbsp olive oil - 1 tbsp red wine vinegar - 1 tbsp basil leaves, chopped   **Fruit:**   - **1 cup green grapes / 1 cup blueberries**   **Iced Tea:**  8 oz unsweetened iced tea |  |
| **Afternoon Snack** | **Yogurt with cereal and granola:**   - ¾ cup nonfat Greek yogurt - ½ cup corn flakes cereal - 2 tbsp granola   **Chocolate:**   - 0.25 oz dark chocolate   **Fruit:**   - **1 cup green grapes / 1 cup frozen blueberries** | **Veggies and crackers with hummus:**   - 3 stalks celery - ¼ cup hummus - 3 whole wheat crackers | **Popcorn and dark chocolate:**   - 1 cup air popped popcorn - 1 oz dark chocolate - ½ c. orange juice |  |
| **Dinner** | **Chicken thighs with naan flatbread and roasted vegetables:**   - 2 oz chicken thighs, skinless, roasted - 1 piece whole wheat naan - 4 tbsp hummus - ½ cup green peppers, chopped - ½ cup zucchini, boiled, sliced - 0.5 tbsp olive oil   **Milk:**   - 8 oz nonfat milk fortified w/ Vitamins A & D | **Salmon over quinoa and veggies/greens:**   - 2 oz salmon fillet, cooked - 1 cup quinoa, cooked - 1.5 cups chopped spinach - 0.5 oz feta cheese - ¼ cup cucumber, sliced - 2 medium asparagus spears, chopped   **Milk:**   - 8 oz nonfat milk fortified w/ Vitamins A & D | **Turkey & black bean chili with a side of cornbread:**   - 1.5 oz ground turkey - ½ cup black beans, canned, low sodium - ¼ cup white onion, diced - 6 oz (~1/2 can) crushed canned tomatoes - ½ cup red bell pepper, chopped - 1 piece cornbread   **Milk:**   - 8 oz nonfat milk fortified w/ Vitamins A & D |  |
| **[Menu Footnote]** | *This menu item follows the Healthy US dietary pattern and features a variety of whole grain and refined grains* | *This menu item follows the Healthy US dietary pattern and features a variety of whole grain and refined grains* | *This menu item follows the Healthy US dietary pattern and features a variety of whole grain and refined grains* |  |
